# Directional Transport Is Mediated by a Dynein-Dependent Step in an RNA Localization Pathway

**DOI:** 10.1371/journal.pbio.1001551

**Published:** 2013-04-30

**Authors:** James A. Gagnon, Jill A. Kreiling, Erin A. Powrie, Timothy R. Wood, Kimberly L. Mowry

**Affiliations:** Department of Molecular Biology, Cell Biology & Biochemistry, Brown University, Providence, Rhode Island, United States of America; Cancer Research UK, United Kingdom

## Abstract

Cytoplasmic RNA localization is a key biological strategy for establishing polarity in a variety of organisms and cell types. However, the mechanisms that control directionality during asymmetric RNA transport are not yet clear. To gain insight into this crucial process, we have analyzed the molecular machinery directing polarized transport of RNA to the vegetal cortex in *Xenopus* oocytes. Using a novel approach to measure directionality of mRNA transport in live oocytes, we observe discrete domains of unidirectional and bidirectional transport that are required for vegetal RNA transport. While kinesin-1 appears to promote bidirectional transport along a microtubule array with mixed polarity, dynein acts first to direct unidirectional transport of RNA towards the vegetal cortex. Thus, vegetal RNA transport occurs through a multistep pathway with a dynein-dependent directional cue. This provides a new framework for understanding the mechanistic basis of cell and developmental polarity.

## Abstract 2

*In vivo* imaging of subcellular RNA localization in *Xenopus* oocytes reveals domains of transport directionality mediated by distinct molecular motors, with dynein providing a directional cue for polarized transport.

## Author Summary

Like traffic on highways, molecular cargos are transported within cells on tracks that are collectively referred to as cytoskeletal networks. RNA molecules are one such cargo, and in many species, the localization of RNAs in egg cells or oocytes is essential for establishing the first asymmetries that are necessary for proper embryo development. RNAs can be actively transported by molecular motors that move cargos along the cytoskeletal tracks, but how such motors are capable of directing cargos to specific destinations within the cell is not yet known. Here we show that two motors, dynein and kinesin—known to carry out transport in opposite directions—are both directly involved in RNA localization in frog oocytes. To understand how these motors can promote directional cargo transport, we developed a system to monitor RNA transport in live oocytes. We find that the motor acting first in the pathway, dynein, is responsible for unidirectional transport. Bidirectional transport, mediated by kinesin, occurs subsequently on cytoskeletal tracks of opposing polarity near the RNA's final destination. Our results suggest a new model for directional transport comprising an initial directional cue that dominates over a later nondirectional step, acting to refine the ultimate cargo distribution.

## Introduction

RNA transport underlies cell and developmental polarity in many organisms. Spatial regulation of gene expression mediated by subcellular RNA localization is required for embryonic axis formation, germ cell specification, and neuronal polarity [1],[2]. While there are several means by which cells can achieve mRNA localization, perhaps the most common of these relies on active transport by molecular motors. In metazoans, kinesin and dynein motor proteins drive transport of RNA and other cargos to the plus- and minus-ends of microtubules, respectively [3]–[5]. In most models of mRNA localization, a single type of motor is bound to the RNA cargo to mediate localization. However, this simple model fails to account for cells that possess microtubule arrays of mixed polarity, or situations where the RNA cargo is capable of binding both plus- and minus-end directed motors. These issues raise the critical question of how RNAs are targeted to the correct subcellular location when they are capable of bidirectional transport.

In *Xenopus* oocytes, localization of Vg1 mRNA to the vegetal cortex during oogenesis is essential for proper germ layer patterning during embryogenesis [6]. Vg1 mRNA encodes a member of the TGF-β growth factor family, and spatially restricted expression of Vg1 is critical for both endoderm and mesoderm specification [7],[8]. Vegetal transport of Vg1 mRNA is directed by a vegetal localization element (VLE) contained within the 3′ UTR [9]. The VLE associates with both sequence-specific RNA-binding proteins [10] and molecular motors, including kinesin-1 and kinesin-2 [11],[12]. However, kinesin motors mediate transport only in the lower half of the vegetal cytoplasm [12], near the RNA's final destination, indicating that vegetal RNA transport must require additional steps and may rely on other molecular motors. Importantly, the mechanisms controlling directionality during RNA transport are poorly understood in most systems.

Regulating the net direction of transport is crucial, both for RNAs and for other cargos such as vesicles and organelles. This important process is not well understood, although recent studies have suggested a range of possible mechanisms. Some models favor regulation at the level of the molecular motor, whereby control of motor number, motor activity, or both is responsible for directional transport [13]–[17]. Other studies have implicated microtubule modifications or asymmetries in microtubule polarity [12],[18],[19]. Support for both classes of models has emerged from studies of RNA localization. For example, RNAs containing localization elements that direct localization to the apical cytoplasm of the *Drosophila* blastoderm embryo recruit more dynein motors than are recruited to nonlocalizing transcripts [14],[20]. This promotes directional transport by favoring processive movement towards the minus ends of microtubules for apically localized RNAs [14],[20]. By contrast, *oskar* RNA localization by the kinesin-1 motor relies on a slight bias in microtubule orientation within the *Drosophila* oocyte for directional transport [19]. Vg1 mRNA transport also depends on microtubule orientation; a subpopulation of microtubules oriented with their plus-ends towards the vegetal cortex emerges coincident with kinesin-1-dependent transport of Vg1 mRNA [12]. Although it is likely that multiple motors coordinate transport of localized RNAs on polarized microtubule networks, the mechanisms by which polarized transport can be controlled by motors of opposing directionality on a dynamic cytoskeleton remain unknown. Moreover, because most studies have relied on tracking individual mRNP particles to determine the rate and direction of transport, our understanding of how the flux of mRNA populations can promote localization to the appropriate destination is far from complete.

To uncover the basis for directional RNA transport in *Xenopus* oocytes we have analyzed the functions of specific molecular motors in vegetal RNA transport. Through biochemical and in vivo interference experiments, we show that cytoplasmic dynein is required for an initial step in the vegetal RNA transport pathway. This is followed by a kinesin-dependent step that ultimately brings the RNA to the cortex. Using in vivo imaging approaches, we measured the rate and direction of RNA movement, revealing discrete regions of transport directionality in the oocyte cytoplasm. While transport near the vegetal cortex is bidirectional, dynein-dependent transport is strongly biased toward the vegetal pole and thus provides the initial directional cue for polarized RNA transport. Our results reveal an unexpectedly complex choreography, with multiple motors moving in several directions, to ensure delivery of RNA cargos to their precise destination.

## Results

### Dynein Plays an Essential Role in Vegetal RNA Localization

**Figure 1:**
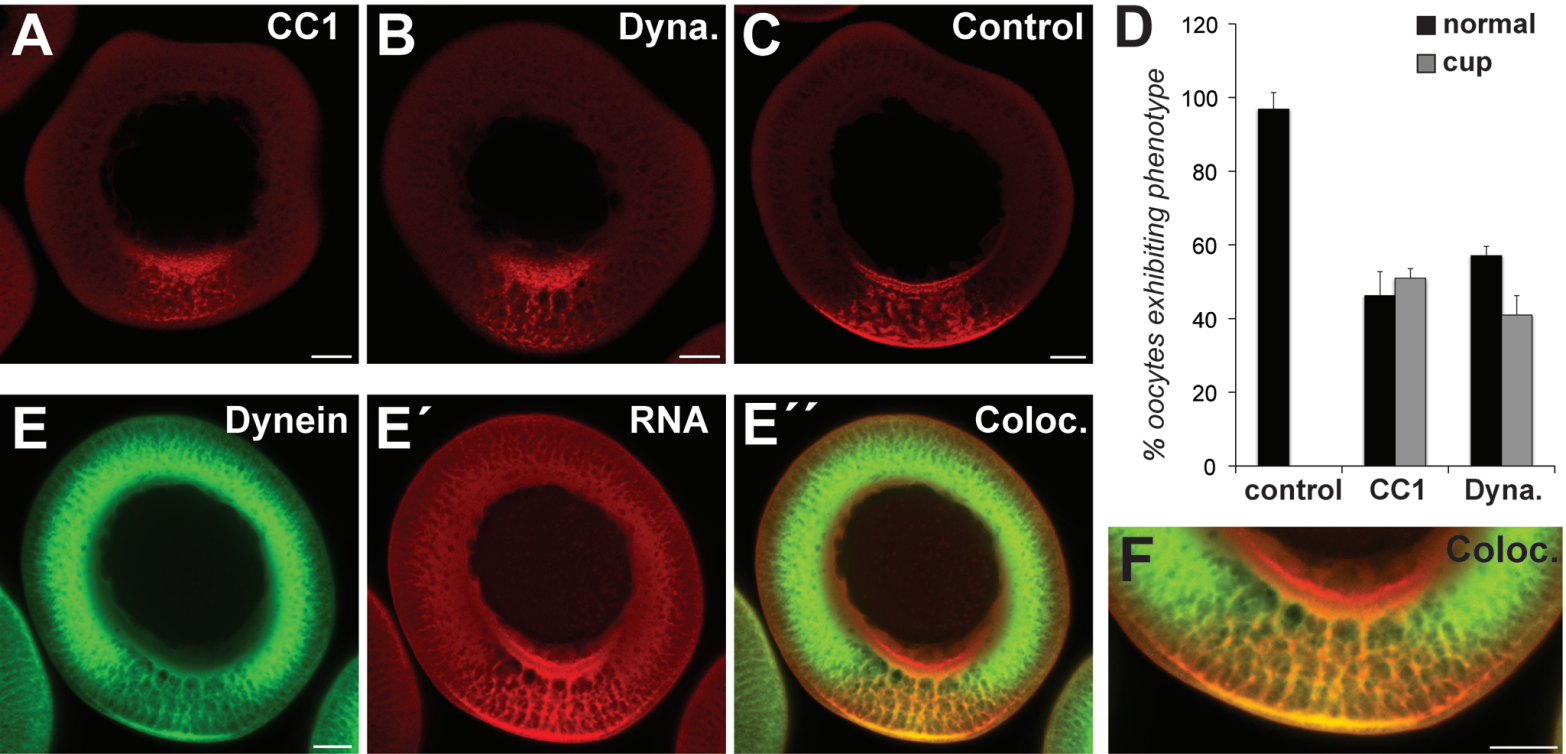
Dynein is required for vegetal RNA localization. (A–C) Fluorescently labeled VLE RNA was microinjected into oocytes expressing (A) p150^Glued^ CC1 domain, (B) p50-dynamitin, or (C) no exogenous protein. (D) Quantification of in vivo interference results (control [control, *n* = 69], p150^Glued^ CC1 [CC1, *n* = 97], and p50-dynamitin [Dyna., *n* = 98]). Black bars indicate normal localization; gray denotes cup accumulation. Error bars indicate standard deviation. (E) Oocytes microinjected with fluorescently labeled VLE RNA were probed with anti-dynein. Shown is a confocal section with dynein in green (E), VLE RNA in red (E′), and co-localization in yellow (E″). (F) Zoomed view of (E″) showing the vegetal cytoplasm. (A–F) Representative confocal images of fixed oocytes are shown, with the vegetal pole towards the bottom. Scale bars, 50 µm.

Transport of RNA to the vegetal cortex of the *Xenopus* oocyte is mediated in part by kinesin motors [11],[12], but other necessary steps in the transport pathway appear to be independent of kinesin. Specifically, kinesin motors have been shown to mediate vegetal RNA transport in the lower vegetal cytoplasm near the vegetal cortex, but not in the upper vegetal cytoplasm [12], leaving open the question of what machinery could mediate transport in that region. While microtubules in stage II–III oocytes are generally oriented with minus ends at the vegetal cortex, a subpopulation is present within the vegetal cytoplasm with plus-ends at the vegetal cortex [12],[21]. Thus, the vegetal cytoplasm contains microtubules of opposing polarities, with both plus- and minus-ends pointing toward the vegetal cortex. Given the mixed population of microtubules present within the vegetal cytoplasm [12], we investigated the role of cytoplasmic dynein in the vegetal RNA transport pathway. Dynein mediates transport to minus ends of microtubules [22] in conjunction with dynactin [23],[24], a distinct protein complex that is required for dynein-dependent transport of vesicles, organelles, spindles, peroxisomes, and mRNAs [4],[5],[22]. To test a potential role for dynein in vegetal RNA transport, we disrupted dynein function in vivo. We used three approaches that have previously been shown to block dynein-dependent transport by disrupting interactions between dynein and dynactin; two relied on overexpression of dynactin components [25],[26], either the CC1 domain of p150^Glued^ (Figure 1A) or dynamitin (Figure 1B), and the third used microinjection of a function blocking dynein antibody (Figure S1). After disrupting dynein function, we microinjected fluorescently labeled VLE RNA to assess effects on mRNA localization. In untreated control oocytes (Figure 1C), VLE RNA undergoing localization adopts a characteristic distribution in the vegetal cytoplasm, typified by a cup of RNA on the vegetal side of the nucleus and a crescent of RNA at the vegetal cortex, with the RNA in the process of localization evident in the vegetal cytoplasm between the cup and cortex (see Figure S2 for a time course of vegetal RNA localization). Disruption of dynein function using all three approaches caused a strong enrichment of VLE RNA in a cup-like region on the vegetal side of the oocyte nucleus and significant loss of RNA accumulation in the lower vegetal cytoplasm and cortex (Figure 1A–B, Figure S1B) relative to VLE RNA localization in control oocytes (Figure 1C–D, Figure S1A). The distribution of microtubules in the vegetal cytoplasm was unaffected by dynein disruption (Figure S3), as was that of dynein itself (Figure S4), suggesting a direct effect on vegetal RNA localization. The observed loss of VLE localization after disruption of dynein function reveals a critical function for dynein in vegetal RNA localization.

**Figure 2:**
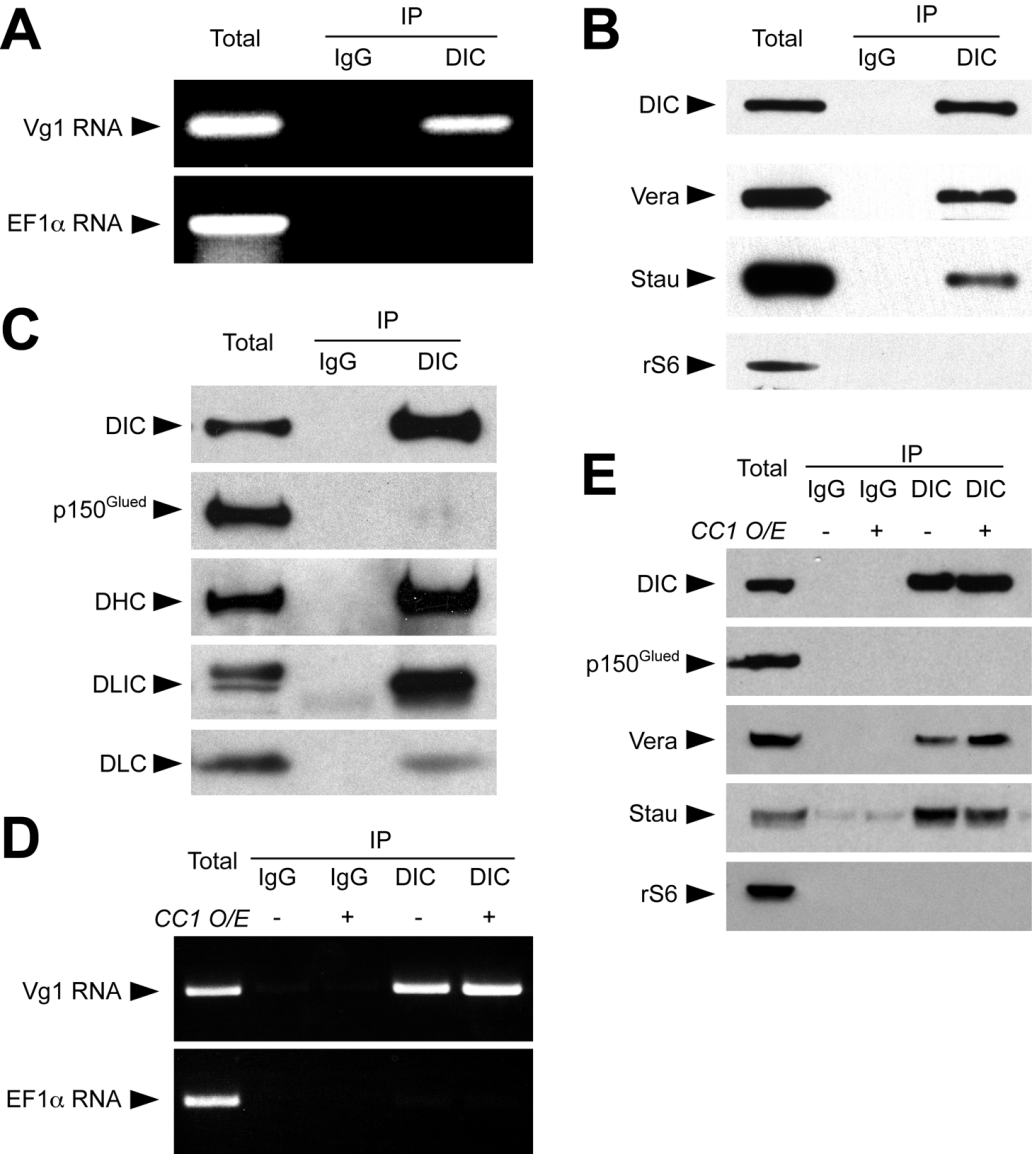
Dynein is associated with Vg1 RNA. (A) Oocyte lysates were immunoprecipitated with nonspecific mouse IgG or mouse anti-dynein intermediate chain (DIC), and bound Vg1 and EF1α RNAs were detected by RT-PCR. (B) Oocyte lysates were immunoprecipitated as in (A) and blotted after SDS-PAGE with antibodies against DIC, Vg1RBP/Vera (Vera), Staufen (Stau), or ribosomal protein S6 (rS6). (C) Oocyte lysates were immunoprecipitated as in (B) and blotted with antibodies for DIC, the p150^Glued^ subunit of dynactin (p150^Glued^), dynein heavy chain (DHC), dynein light intermediate chain (DLIC), and dynein light chain (DLC). (D) Control oocytes (−) or oocytes expressing p150^Glued^ CC1 (+) were lysed and immunoprecipitated as in (A); bound Vg1 and EF1α RNAs were detected by RT-PCR. (E) Lysates from control (−) or p150^Glued^ CC1 expressing (+) oocytes were immunoprecipitated as in (B) and blotted with antibodies for DIC, p150^Glued^, Vera, Stau, and rS6.

To address whether dynein plays a direct role in vegetal RNA transport, we tested whether dynein is specifically associated with Vg1 RNA. We first performed immunofluorescence for dynein in oocytes that were microinjected with fluorescent VLE RNA. Dynein (Figure 1E) and VLE RNA (Figure 1E′) are colocalized at the cortex and throughout the vegetal cytoplasm (Figure 1E″–F). In addition, immunoprecipitation of dynein complexes (Figure 2A) using a dynein intermediate chain (DIC) antibody specifically recovered Vg1 mRNA but not a highly abundant control RNA, EF1α. Immunoprecipitation of dynein complexes (Figure 2B) also recovered known components of the Vg1 mRNP [10], including Vera and Staufen, but perhaps surprisingly, did not recover the dynactin component, p150^Glued^ (Figure 2C). Although the dynactin complex has been suggested to be required for dynein/cargo association, dynactin has also been shown to regulate dynein transport by increasing processivity and modulating interaction with microtubules [27]. Importantly, recruitment of cargos to dynein can be dynactin-independent, although transport still requires dynactin [28]–[31]. Consistent with a dynactin-independent mode of cargo binding, disruption of dynactin by CC1 overexpression does not abolish dynein/Vg1 mRNP association (Figure 2D,E), yet blocks vegetal RNA transport (Figure 1).

### Dynein Acts Prior to Kinesin-1 in the Vegetal RNA Transport Pathway

**Figure 3:**
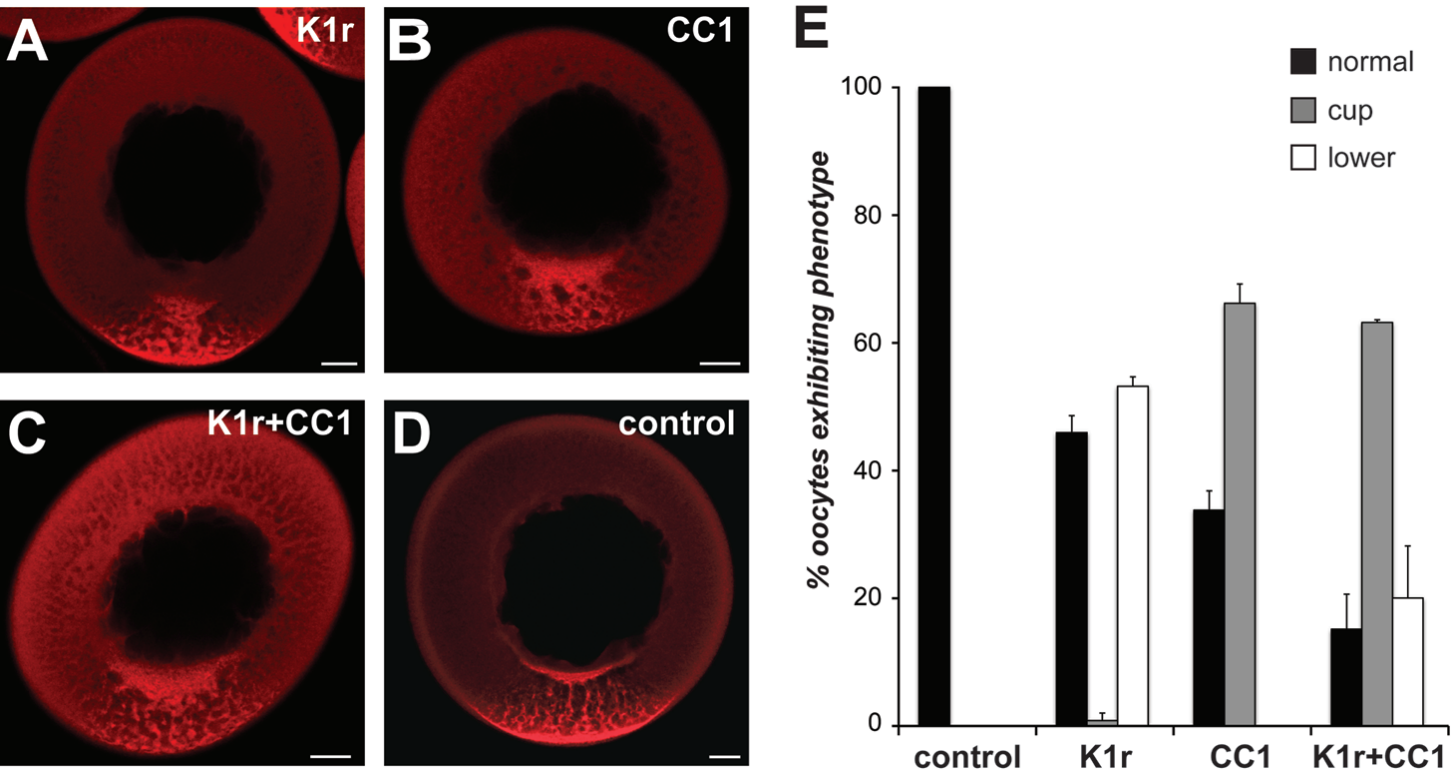
Dynein and kinesin mediate distinct steps in vegetal RNA transport. (A–D) Fluorescently labeled VLE RNA was microinjected into oocytes expressing (A) kinesin-1 rigor mutant (K1r), (B) p150^Glued^ CC1 (CC1), (C) both K1r and CC1, or (D) no exogenous protein. Representative oocytes are shown with the vegetal pole towards the bottom. Scale bars, 50 µm. (E) Quantification of in vivo interference results for oocytes expressing no exogenous protein (control, *n* = 167), kinesin-1 rigor (K1r, *n* = 159), CC1 domain of p150^Glued^ (CC1, *n* = 209), and both K1r and CC1 (*n* = 208). Black bars indicate normal localization, gray denotes cup accumulation, and white bars indicate accumulation of RNA in the lower vegetal cytoplasm. Error bars indicate standard deviation.

The phenotype observed upon dynein disruption, accumulation of VLE RNA in the cup region on the vegetal side of the nucleus (Figure 1A–B), is quite distinct from that previously observed upon expression of a kinesin-1 rigor mutant [12]. This dominant negative mutant locks cargo onto microtubules at the site of kinesin binding [32] and results in accumulation of VLE RNA in the lower vegetal cytoplasm ([12], Figure 3A). Moreover, the dynein-disruption phenotype is also distinct from that occurring upon inhibition of kinesin-1 by overexpression of kinesin-1 heavy chain lacking its motor domain (KHCΔm), which results in no detectable vegetal localization (Figure S5). The distinct RNA distributions after either dynein or kinesin-1 disruption (Figure 3A,B) suggest that dynein and kinesin could mediate sequential steps in vegetal RNA transport. To order these motors in the RNA transport pathway, we disrupted the function of both motors simultaneously in oocytes (Figure 3C). We observed that accumulation of VLE RNA in the perinuclear cup after disruption of dynein function by CC1 overexpression was unaffected by kinesin-1 rigor expression, while the kinesin-1 rigor phenotype of accumulation in the lower vegetal cytoplasm was significantly reduced (Figure 3E). The predominance of the dynein-disruption phenotype indicates that dynein functions upstream of kinesin-1 in the transport pathway. Yet how these motors, known to move in opposing directions on microtubules, might carry out transport of Vg1 RNA to the vegetal cortex is unclear, and insight into directionality is impossible to attain using standard fixed cell imaging technologies.

### RNA Transport Dynamics Are Revealed by in Vivo Imaging

**Figure 4:**
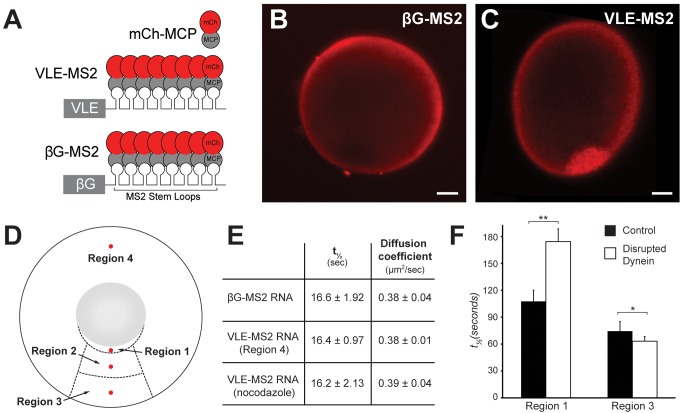
Live imaging of RNA localization reveals RNA transport dynamics. (A) Diagram of VLE RNA (VLE-MS2) and nonlocalizing β-globin RNA (βG-MS2) tagged with multimerized MS2 binding sites, which recruit MS2 coat protein fused to mCherry (mCh-MCP). (B) Oocytes expressing mCh-MCP and injected with βG-MS2 RNA exhibit uniform cytoplasmic fluorescence. (C) Oocytes expressing mCh-MCP and injected with VLE-MS2 RNA exhibit strong vegetal fluorescence (red). (B–C) Images of live oocytes are shown, with vegetal poles towards the bottom; scale bars, 20 µm. (D) Diagram of oocyte showing regions used for analysis: cup region immediately adjacent to the nucleus on the vegetal side (Region 1), the upper vegetal cytoplasm (Region 2), the lower vegetal cytoplasm (Region 3), and the animal hemisphere (Region 4). The 5 µm circular regions for FRAP are indicated in red and are spatially defined in Materials and Methods. (E) Calculated half times of recovery and diffusion coefficients from FRAP analysis. βG-MS2 RNA mobility was measured in Regions 2–4 and VLE-MS2 RNA mobility was measured in Region 4. After nocodazole treatment, VLE-MS2 RNA mobility was measured in Regions 2 and 3. ± indicates standard error of the mean. (F) Averaged halftimes (t_½_) of recovery for indicated regions in control oocytes (black bars) or oocytes with dynein function disrupted by expression of CC1 (white bars). Control (Region 1, *n* = 10; Region 3, *n* = 10), Disrupted dynein (Region 1, *n* = 21; Region 3, *n* = 22). Error bars show standard error of the mean. Half times of recovery (t_1/2_) and diffusion coefficients were calculated as described in Materials and Methods. The *p* values were generated using a two-tailed unpaired Student's *t* test; ** *p* = 0.0054, * *p* = 0.299.

To define specific roles for molecular motors in directional RNA transport, we developed a live imaging system for *Xenopus* oocytes by adapting a method first established for imaging RNA transport in yeast [33]. As depicted in Figure 4A, a fluorescent protein, mCherry (mCh, [34]) in this case, is tethered to the RNA of interest by exploiting a strong binding interaction between the MS2 bacteriophage coat protein (MCP) and a 21-nucleotide RNA hairpin [35]. Injection of a nonlocalized RNA tagged with MS2 hairpins (βG-MS2) into live *Xenopus* oocytes expressing mCh-MCP produced a signal that was uniform throughout the cytoplasm (Figure 4B). By contrast, live oocytes expressing mCh-MCP injected with VLE-MS2 RNA exhibited a strong signal at the vegetal pole (Figure 4C), demonstrating that tethering multiple fluorescent proteins to RNA can be used to monitor RNA localization in *Xenopus* oocytes.

To measure RNA transport in vivo, we used Fluorescence Recovery After Photobleaching (FRAP, [36]). The large size of the *Xenopus* oocyte (∼300 µm diameter at stage II; [37]) allowed us to assess RNA mobility in multiple regions of the vegetal cytoplasm: in the cup region adjacent to the nucleus (Figure 4D, Region 1), in the upper vegetal cytoplasm (Region 2), in the lower vegetal cytoplasm (Region 3), and in the animal hemisphere cytoplasm (Region 4). FRAP analysis showed that the mobility of VLE-MS2 RNA in the vegetal cytoplasm (t_1/2_ = 86.7±6.72 s) is significantly lower than that of a nonlocalizing βG-MS2 RNA (t_1/2_ = 16.6±1.92 s). Yet the mobility of VLE-MS2 RNA outside of the vegetal cytoplasm (Figure 4D, Region 4) is similar (t_1/2_ = 16.4±0.97 s) to that of βG-MS2 RNA. The calculated diffusion coefficients outside of the vegetal cytoplasm (Figure 4E) are consistent with previously published reports of RNA diffusion rates [38],[39], suggesting that VLE RNA can diffuse freely outside of the vegetal cytoplasm. The behavior of VLE RNA in the vegetal cytoplasm is dependent on microtubules, as VLE RNA mobility is similar to βG-MS2 RNA after disruption of microtubules by nocodazole treatment (t_1/2_ = 16.2±2.13 s). Since the FRAP experiments assess the mobility of the entire population of MS2-tagged RNAs in a given region, these results indicate that the majority of VLE-MS2 RNAs in the vegetal cytoplasm are not moving rapidly at any given time. Moreover, the reduced mobility of VLE RNA in the vegetal cytoplasm is likely due to interaction of the RNA with the microtubule cytoskeleton, presumably through an interaction with molecular motors.

To assess the effects of dynein disruption in live oocytes, we performed FRAP in the vegetal cytoplasm in oocytes overexpressing dynamitin and in control oocytes. We carried out FRAP in Regions 1 and 3, but not Region 2, due to enlargement of the cup region in oocytes expressing dynamitin (Figure 1B). As shown in Figure 4F, dynein disruption significantly slowed RNA mobility in Region 1, but had no effect on mobility in Region 3. These results suggest that dynein is required to move RNA out of the cup region towards the vegetal cortex and further indicate that RNA movement in the lower vegetal cytoplasm does not depend on dynein.

### Distinct Regions of Transport Directionality Are Controlled by Dynein and Kinesin-1

**Figure 5:**
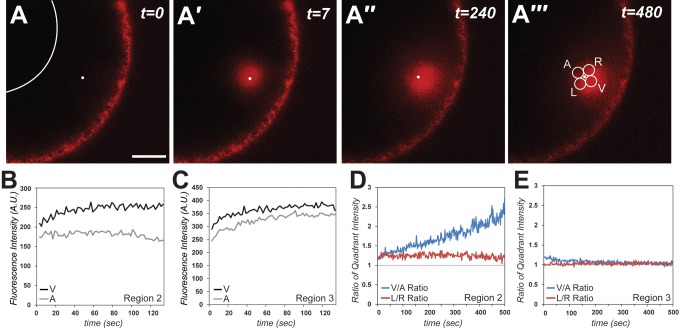
Distinct regions of RNA transport directionality. Oocytes expressing PA-mCh-MCP were microinjected with VLE-MS2 RNA. (A) Prior to activation of PA-mCh-MCP in live oocytes (t = 0), minimal fluorescence is observed. The activation point is shown by the small white dot and the oocyte nucleus is outlined in white. Scale bar, 20 µm. (A′) By 7 s after activation of PA-mCh-MCP, robust fluorescence (red) is evident at and around the activation point (white dot). (A″–A′″) By 240–480 s after activation, PA-mCh-MCP tethered to RNA can be visualized asymmetrically around the activation point. (A′″) The four collection quadrants are indicated by white circles surrounding the activation point: V and A indicate the collection quadrants on the vegetal and animal sides of the activation point, respectively. L and R indicate the collection quadrants on the left and right sides. (B–C) After activation of PA-mCh-MCP in (B) the upper vegetal cytoplasm (Region 2) or (C) the lower vegetal cytoplasm (Region 3), corrected fluorescence intensities in the V (black) and A (grey) quadrants were plotted over time. (D, E) The ratios of V∶A (red) and L∶R (blue) intensities for oocytes activated in (D) the upper vegetal cytoplasm (Region 2) and (E) the lower vegetal cytoplasm (Region 3) were plotted over time.

Directionality is crucial to understanding the mechanisms controlling asymmetric RNA transport, and roles for motors that move in opposing directions on microtubules complicate this issue. We have previously described a subpopulation of microtubules, present at the vegetal pole during mid-oogenesis, which are oriented with plus ends at the cortex [12]. This subpopulation is superimposed over a microtubule network present throughout the oocyte cytoplasm, which is oriented with minus ends toward the cortex [21]. Thus, microtubules are polarized with plus ends at the nucleus and minus ends pointed toward the cortex in the upper vegetal cytoplasm, while in the lower vegetal cytoplasm the microtubule array is mixed, with microtubules oriented in both directions [12]. To test whether transport directionality differs between these regions, we extended our live imaging system by incorporating a photoactivatable form of mCherry (PA-mCh-MCP), which is nonfluorescent until laser stimulation [40]. Activation of the fluorophore, bound to VLE-MS2 RNA, in specific regions of the oocyte allows RNP transport directionality to be tracked in defined regions of the cytoplasm. Expressed in vivo, PA-mCh-MCP was nonfluorescent (Figure 5A, t = 0) until after activation (Figure 5A′, t = 7 s). To discern any potential asymmetry in transport, we tracked RNA movement at time points after activation (Figure 5A′–A′″) by measuring the fluorescence in four collection quadrants—left (L), right (R), animal (A), and vegetal (V)—surrounding the activation point (Figure 5A′″, white circles). After activation in Region 2 (Figure 5B), fluorescence intensity increases in the vegetal collection quadrant (black line), while fluorescence in the animal quadrant (gray line) remains relatively constant. By contrast, fluorescence intensities increase in both the animal and vegetal collection quadrants after activation in Region 3 (Figure 5C). Control experiments show that for oocytes injected with nonlocalizing β-globin RNA (βG-MS2), fluorescence intensity decreases after activation (Figure S6A), consistent with diffusive movement. To determine whether VLE RNA movement (Figure 5A–C) might represent active transport on microtubules, we disrupted microtubules by treatment with nocodazole in oocytes injected with VLE-MS2 RNA. In both the upper (Figure S6B) and lower (not shown) vegetal cytoplasm, fluorescence intensity decreased rapidly upon activation following microtubule disruption and was similar to results observed in the animal hemisphere cytoplasm (Region 4, Figure S6C). Thus, only diffusive movement is apparent after microtubule disruption, suggesting that RNA movement in the vegetal cytoplasm results from active transport on microtubules. To quantify transport directionality in the AV axis, we determined the ratio of intensities in the V versus A collection quadrants over time (Figure 5D,E). V∶A ratios at or near 1 indicate no bias in transport directionality, while values greater than 1 represent vegetally directed transport. After activation in the upper vegetal cytoplasm (Region 2, Figure 5D), the V/A quadrant signal intensity (blue) increases over time, indicating directional transport toward the vegetal pole; no bias in left-right transport (red) is detectable. Averaged V/A intensities after 8 min of RNA transport (Figure S7A) demonstrate significant bias in vegetal (64%) versus animal (36%) quadrant signal, again supporting directed vegetal transport in Region 2. By contrast, activation in the lower vegetal cytoplasm (Region 3) exhibits no trend in either direction over time (Figure 5E), and no bias between vegetal and animal transport (47% vegetal quadrant signal versus 53% animal quadrant signal; Figure S7B), suggesting bidirectional transport in Region 3.

**Figure 6:**
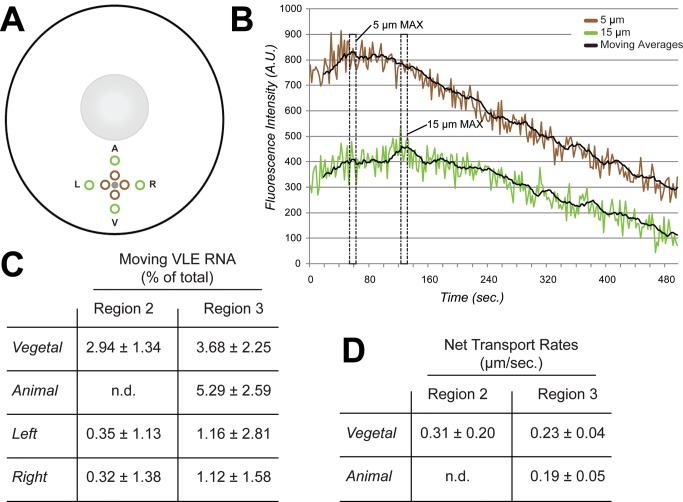
Analysis of net transport after photoactivation. Oocytes expressing PA-mCh-MCP were injected with VLE-MS2 RNA and photoactivated as in Figure 5. (A) Collection windows were defined at 5 µm (brown) and 15 µm (green) from the site of photoactivation (gray) in all four directions: Animal (A), Vegetal (V), Left (L), and Right (R). (B) Data from an oocyte activated in the lower vegetal cytoplasm and collected on the vegetal side of the activation point are shown. Fluorescence intensity collected in the defined windows is plotted over time, along with a moving average trendline (period = 10). The timepoints of intensity maxima (MAX) for the 5 µm (t_1_) and 15 µm (t_2_) collection windows are indicated by dashed boxes. (C) The percentages of VLE-MS2 RNA moving in the Vegetal, Animal, Left, and Right directions were calculated by measuring change in fluorescence intensity in collection windows 5 µm and 15 µm away from the activation point (see Materials and Methods for details). Standard deviation is indicated by ±, *n* = 5. RNA movement in the animal direction could not be detected (not detected, n.d.) in Region 2 because intensity maxima in the collection windows were simultaneous, suggesting no transport in this direction within Region 2. (D) Transport rates were calculated for the motile fraction of VLE-MS2 RNA in Regions 2 and 3 by identifying time points (t_1_, t_2_) of peak fluorescence intensity in collection windows 5 µm and 15 µm away from the activation point (see Materials and Methods for details). The calculated rates of net RNA transport are shown, with error (±) given as standard deviation, *n* = 5.

Our photoactivation experiments suggest a directional bias to transport in the upper vegetal cytoplasm and bidirectional transport in the lower vegetal cytoplasm. However, the lack of directional bias in the lower vegetal cytoplasm could alternatively be due to no movement of the RNA in that region. To address this issue, we quantified the motile fraction of the RNA population by identifying and tracking profiles of fluorescence intensity during RNA transport at 5 µm and 15 µm from the site of photoactivation (Figure 6A,B). In agreement with the FRAP results (Figure 4), which suggested that the majority of VLE RNA was not moving rapidly in the vegetal cytoplasm, we find (Figure 6C) that only ∼3–5% of the RNA is moving in either region of the vegetal cytoplasm during the ∼1-min time frame between t_1_ and t_2_ (see Figure 6B). Importantly, only vegetally directed movement is detected in the upper vegetal cytoplasm, while in the lower vegetal cytoplasm, VLE RNA is moving in both directions (Figure 6C). We do not observe a significant fraction of RNA to be moving in the left or right direction in either Region 2 or 3 (Figure 6C), nor any directional bias in left/right RNA distribution in any region (Figure S7). Tracking the fluorescence intensity profiles (Figure 6B) also enabled us to determine net transport rates in specific regions of the vegetal cytoplasm (Figure 6D). In the upper vegetal cytoplasm, net RNA movement (at ∼0.3 µm/s) is observed only toward the vegetal cortex, while in the lower vegetal cytoplasm RNA movement is at similar rates in both animal and vegetal directions (Figure 6D). Taken together, our live cell imaging results support a model for vegetal RNA transport in which kinesin-dependent transport in the lower vegetal cytoplasm is bidirectional, while dynein-dependent transport in the upper vegetal cytoplasm is strongly biased toward the vegetal cortex.

## Discussion

Although molecular motors are known to play important roles in RNA transport, ordering individual steps into a coherent pathway within a single cell has been a major challenge. We have uncovered surprising complexity in the RNA transport pathway that brings RNAs critical for germ layer patterning to the vegetal cortex of the *Xenopus* oocyte. Rather than a single type of motor driving RNA transport in a single direction, we find that distinct motors direct vegetal transport in defined domains of the oocyte cytoplasm. Specifically, the minus-end-directed motor dynein is responsible for RNA transport in the upper vegetal cytoplasm, while transport in the lower vegetal cytoplasm relies on plus-end directed kinesin motors. The complementary phenotypes we obtained upon dynein and kinesin-1 disruption indicate roles for these motors in distinct transport steps, and the predominance of the dynein phenotype upon simultaneous disruption of both motors (Figure 3E) demonstrates that dynein functions upstream of kinesin in the transport pathway. Although dynein does not function in transport of Vg1 mRNA in the lower vegetal cytoplasm, as evidenced by FRAP experiments showing that dynein disruption does not affect RNA mobility in that region (Figure 4F), we propose that dynein remains part of the Vg1 mRNP as dynein is colocalized with VLE RNA in the lower vegetal cytoplasm and at the oocyte cortex (Figure 1F, Figure S8). It is possible that dynein also plays an indirect role here by recruiting kinesin motors to the Vg1 RNP, or by localizing the motors themselves. Further, in light of reports that dynein can transition from motor activity to function as a stable anchor for localized RNAs [41],[42], dynein may play a similar role in vegetal RNA localization. The molecular mechanism of the association of dynein with localized RNA is also intriguing. Dynein is able to associate with the Vg1 mRNP after disruption of the dynactin complex (Figure 2D,E), although transport of Vg1 mRNA by dynein is dependent on dynactin (Figure 1A–D). This suggests that dynactin is required in this system for motor activity but not for mRNA cargo selection, in agreement with several recent studies [28]–[31]. As has been proposed for transport of RNAs by dynein in the *Drosophila* embryo [28], RNA binding proteins could mediate a direct interaction between Vg1 mRNA and the dynein motor.

**Figure 7:**
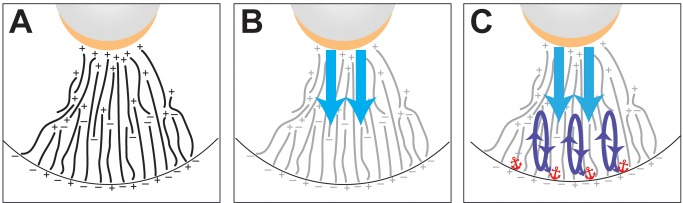
Model for vegetal RNA localization. The vegetal cytoplasm is depicted, with the vegetal cortex at the bottom. The oocyte nucleus is shown in gray and the perinuclear cup is indicated in gold. (A) The oocyte microtubules are shown in black with orientation indicated by plus and minus. The proposed arrangement of microtubules is based on the appearance of a subpopulation of microtubule plus-ends at the vegetal cortex following breakdown of the mitochondrial cloud [12], which has been proposed to contain a microtubule organizing center [52]. (B) Vg1 mRNA enriched at the perinuclear cup is first transported by the dynein molecular motor in the upper vegetal cytoplasm in an initial highly directional step toward the vegetal cortex (blue). Microtubules are shown in grey. (C) Repeated cycles of bidirectional transport dependent on kinesin molecular motors occur in the lower vegetal cytoplasm (purple), until Vg1 mRNA exits the transport cycle by becoming anchored at the vegetal cortex (red).

Our live imaging experiments reveal distinct kinetics and directionality for RNA transport in different regions of a single cell. For the first time in the *Xenopus* oocyte, we have been able to measure rates of RNA transport. Although quantification of transport rates for RNP particles is available in a number of systems (for example, [14],[19],[39]), the transport rates we have determined provide new information, as it is the net transport of an RNA population that determines how quickly a localized RNA is restricted at its destination. Vg1 mRNA localization takes a surprisingly long time; injected RNA is first enriched in the perinuclear cup region within a few hours (Figure S2A), but takes more than 24 h to become fully localized to the vegetal cortex, a distance of ∼100 µm (Figure S2C). Why does this take so long? Our results suggest two possible reasons. First, we find that while the transport rates we measure (0.2–0.3 µm/s) are consistent with rates of motor-driven transport [43], only a small fraction (3–5%) of Vg1 mRNA in the vegetal cytoplasm is moving at any given time, while the majority remains relatively static, in association with the cytoskeleton. Resolving this traffic jam of stalled cargo will dramatically increase the time needed to complete transport of the entire RNA population. Second, transport is bidirectional near the vegetal cortex, which indicates that not all of the RNA that transported vegetally will be immediately anchored at the vegetal cortex. As depicted in our model (Figure 7), this suggests that the kinesin-dependent transport step may represent a cycle that must be repeated until all the RNA is successfully anchored at the cortex.

Directionality is key to achieving polarized transport, and our proposed vegetal RNA localization pathway, in which dynein-dependent transport in the upper vegetal cytoplasm precedes kinesin-dependent transport in the lower vegetal cytoplasm, raises important questions regarding directionality given the previously described mixed microtubule polarity in the lower vegetal cytoplasm ([12], Figure 7A). Our experiments (Figures 5–6) reveal domains of RNA transport directionality under control of distinct molecular motors. These results contrast with a recent study of *oskar* mRNA localization in *Drosophila* oocytes, in which dynein-dependent deposition of *oskar* mRNA from the accessory nurse cells into the oocyte precedes kinesin transport to the posterior on a weakly polarized cytoskeleton [19]. While we have placed dynein and kinesin in a similar order in Vg1 transport, our work demonstrates a requirement for both motors in sequential transport steps within a single cell, the oocyte. We have shown that in the *Xenopus* oocyte, kinesin-dependent transport in the lower vegetal cytoplasm traffics on microtubules with plus-ends both away from and toward the vegetal cortex [12], although we cannot rule out a slight bias in transport in either direction. Most importantly, transport in the upper vegetal cytoplasm of the oocyte requires dynein and is strongly biased toward the vegetal cortex, providing a directional cue for vegetal transport. We propose (Figure 7) that, after motor-independent accumulation in the perinuclear cup region, unidirectional dynein-based transport funnels RNA toward the vegetal cortex. Although kinesin transports RNA on a bidirectional array of microtubules near the vegetal cortex, RNA that reaches the cortex has the potential to be captured and stably anchored. RNA that is not anchored can be transported back toward the animal hemisphere, but once it reaches the upper vegetal cytoplasm, the RNA will again be funneled toward the cortex. This keeps the RNA out of the animal hemisphere cytoplasm, where its expression is deleterious to the embryo [8], and after multiple iterations should result in complete localization of the RNA at the vegetal cortex. Such a multistep pathway, relying on the sequential action of motors with opposing polarities, can serve to refine the ultimate distribution of the cargo. It is likely that this new model for the establishment of directional cargo transport may be broadly applicable to polarized transport mechanisms in other cells and systems.

## Materials and Methods

### Ethics Statement

All animal work was conducted according to relevant national and international guidelines.

### Cloning

The CC1 domains of chicken p150^Glued^ (a gift from T. Schroer [26]; GenBank No. NM_001031367), mouse p50/dynamitin (a gift from R. Vallee [25]; GenBank No. NM_027151), mCherry (a gift from R. Tsein [34]; GenBank No. AY678264), PAmCherry1 (a gift from V. Verkushka [40]; GenBank No. 3KCT_A), and MS2 Coat Protein (a gift from R. Singer [33]; GenBank No. NP_040648) were amplified and subcloned into pSP64TSN [44] to create pSP64TSN-CC1, pSP64TSN-Dynamitin, pSP64TSN-mCherry, pSP64TSN-MCP-mCherry, and pSP64TSN-MCP-PAmCherry1 for in vitro transcription. Chimeric VLE-MS2 and βG-MS2 constructs were prepared by subcloning VLE [9] or *Xenopus* β-globin [45] sequences with 24 multimerized MS2 binding sites (a gift from R. Singer [33]) into pSP73 to create pSP73-βG-MS2 and pSP73-VLE-MS2.

### RNA Synthesis, Microinjection, and Oocyte Culture

For in vivo interference, kinesin-1 rigor [12], kinesin-1 lacking the motor domain [12], p50-dynamitin [25], and CC1 [26] RNAs were transcribed using mMESSAGE mMACHINE (Ambion) and diluted to 250–500 nM for microinjection. Fluorescently labeled RNA was transcribed in reactions containing 50 µM Chromatide Alexa Fluor 546-14-UTP (Invitrogen) as previously described [12],[46] and diluted to 50 nM. Microinjected stage III *Xenopus* oocytes were cultured in oocyte culture medium (OCM) [50% L15 medium (Sigma-Aldrich), 15 mM HEPES (pH 7.6), 1 mg/ml insulin (Sigma-Aldrich), 100 mg/ml gentamicin (Gibco), 50 U/ml nystatin (Gibco), 50 U/ml penicillin (Gibco), 50 mg/ml streptomycin (Gibco)] at 18°C for 8–16 h as previously described [46]. To depolymerize microtubules, oocytes were treated with 10 µg/ml nocodazole (Sigma-Aldrich) as previously described [12].

### Immunoprecipitation and Protein Blotting

Oocyte cell lysates were made in 10 mM HEPES pH 7.4, 100 mM potassium acetate, 10 mM magnesium acetate, 5 mM EGTA, 0.1 M sucrose, 1 mM DTT, 0.4 mM Pefabloc SC (Sigma-Aldrich), 0.1% NP-40, 1 U/ml RNasin (Promega), 0.1 µg/ml leupeptin, 0.1 µg/ml antipain, and 0.1 µg/ml trypsin inhibitor. After centrifugation at 10,000× *g* for 10 min, lysates were precleared with mouse IgG-agarose beads (Sigma) before overnight incubation with mouse IgG (Sigma) or mouse anti-dynein intermediate chain antibody (DIC 74.1, Abcam) prebound to Protein A-Sepharose beads (Millipore). After washes, bound samples were reserved for protein blotting, or samples were sequentially treated with DNase and proteinase K before RT-PCR using primers for Vg1 and EF1α, as previously described [12]. For protein blotting, anti-dynein (DIC 74.1, Abcam), anti-p150^Glued^ (Abcam), anti-Vera [47], anti-Staufen [48], anti-dynein heavy chain (a gift from M. Koonce [49]), anti-dynein light intermediate chain (a gift from V. Allan [50]), anti-dynein light chain (Abcam), and anti-rS6 (Cell Signaling) were used at 1∶1,000.

### In Vivo Interference

Oocytes were microinjected with purified function-blocking antibodies (mouse anti-dynein intermediate chain [DIC 70.1, Abcam]) or RNAs (at 250–500 nM) encoding kinesin-1 rigor [12], kinesin-1 lacking the motor domain [12], p50-dynamitin [25], or the CC1 domain of p150^Glued^ [26]. After culture for 2 h (antibodies) or 16 h (RNAs), oocytes were injected with fluorescently labeled VLE RNA and cultured for 8 h. Oocytes were fixed and cleared as previously described [46] before imaging on a Zeiss LSM510 confocal microscope. VLE localization in each oocyte was scored as “normal,” enriched in the “cup” on the vegetal side of the nucleus, enriched in the “lower” half of the vegetal cytoplasm, or “no localization.”

### Immunofluorescence and Confocal Microscopy

Oocytes were permeabilized with 50 µg/ml proteinase K for 3–6 min, fixed in 3.7% formaldehyde for an hour, blocked in 2% BSA and 2% goat serum, and incubated overnight with purified mouse anti-kinesin 1 (SUK4, DSHB) and/or anti-dynein (DIC 74.1, Abcam) antibodies, both at 1∶100 dilution. Oocytes were then washed with PBT (PBS [pH 7.4], 0.2% BSA, 0.1% Triton X-100 [Roche]) and incubated overnight with isotype-specific mouse Alexa-633 (IgG_2b_, DIC 74.1) and Alexa-594 (IgG_1_, SUK4) fluorescent secondary antibodies (Invitrogen), washed with PBT, dehydrated, and stored in anhydrous methanol at −20°C. Oocytes were imaged on either a Zeiss LSM510 or LSM710 confocal microscope as previously described [46].

### Live Cell Imaging

After screening a panel of fluorescent proteins, mCherry ([34]; mCh) was chosen as the best candidate for live cell imaging in *Xenopus* oocytes because of its strong emission at 610 nm, where the autofluorescence of the oocyte cytoplasm is minimal (unpublished data). Stage III albino oocytes were injected with 2 nl of either 250 nM mCh-MCP mRNA or 250 nM PA-mCh-MCP mRNA and incubated overnight in OCM to allow protein expression. Oocytes in some cases were incubated in 10 µg/ml nocodazole to depolymerize microtubules (as in [12]), and subsequently injected with 2 nl of either 250 nM VLE-MS2 RNA or βG–MS2 RNA. After culture for 8 h, live oocytes were mounted in fluorodishes (WPI Inc.) in OCM containing 1% low melting temperature agarose (Sigma-Aldrich) to orient each oocyte for imaging in desired regions (Regions 1–4).

### Fluorescence Recovery After Photobleaching

FRAP analyses were carried out using a Zeiss LSM 510 Meta Confocal Laser Scanning Microscope equipped with a 40× water correction C-Apochromat objective. Regions for FRAP were identified for each oocyte as follows: Region 1 was 15 µm into the vegetal cytoplasm from the germinal vesicle, Region 2 was 50 µm into the vegetal cytoplasm from the germinal vesicle, Region 3 was 25 µm into the vegetal cytoplasm from the vegetal cortex, and Region 4 was 25 µm into the animal cytoplasm from the animal cortex. Within each region, a 5 µm circular region of interest (ROI) was bleached using the 405, 488, 561, and 633 laser lines at 100%. Fluorescence recovery was monitored at 5-s intervals to track VLE-MS2 recovery and 1-s intervals for βG-MS2. FRAP data were fit as previously described [51] to a single exponential rise to maximum model, using the equation: 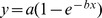, where *a* is the end value of the recovered intensities and *b* is the rate constant. Curve fitting was performed using SigmaPlot 11 software. Half times of recovery (t_1/2_) were calculated using the previously determined rate constant (*b*) in the following equation: 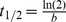. Diffusion coefficients (D) were calculated from the t_1/2_ values by fitting to a simple diffusion model [51] using the following formula: 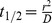, where *r* is the radius of the FRAP ROI.

### Live Cell Photoactivation

A Zeiss LSM 710 Confocal Laser Scanning Microscope equipped with a 40× water correction C-Apochromat objective was used to acquire images. PA-mCh-MCP [40] was activated in a 0.8 µm circular ROI using the 405 laser in Regions 2 and 3 as previously described. Fluorescence intensity was monitored in four 10 µm diameter collection quadrants (A, V, L, and R) surrounding the activation ROI, and collected at 2-s intervals for ∼8 min. Fluorescence intensity before photoactivation was subtracted from postactivation intensities for each collection quadrant to correct for autofluorescence. To evaluate transport directionality, ratios (V∶A or L∶R) of corrected quadrant intensity values were calculated and plotted over time.

### RNA Mobility and Rates of Transport

RNA movement was calculated by defining 5 µm diameter collection windows at 5 µm and 15 µm distances left, right, animal, or vegetal from the site of activation. The fluorescence intensities from each collection window were plotted over time (see Figure 6) to identify the time of intensity maximum in each collection window (t_1_, t_2_). Change in fluorescence intensity within the collection windows was measured in order to quantify the population of moving RNA using the following formulas:
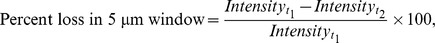
and
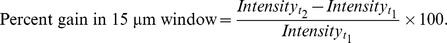
The percent loss in the 5 µm windows and the percent gain in the 15 µm windows were averaged to generate the percentages of motile VLE RNA. Transport rates for the fraction of motile RNA were calculated by dividing the distance between the two collection windows by the time between intensity maxima using the following formula: Transport rate 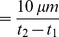.

## Supporting Information

Figure S1 Dynein antibody injection blocks vegetal RNA localization. Oocytes were injected with either (A) control IgG (Sigma) or (B) function-blocking [53] dynein-specific antibodies (DIC 70.1, Abcam). After culture for 2 h, oocytes were injected with fluorescently labeled VLE RNA and cultured for 8 h. Oocytes were fixed and imaged by confocal microscopy. Representative confocal images are shown, with the vegetal pole toward the bottom. Scale bars, 50 µm.
(TIF)

Figure S2 Time course of vegetal RNA localization. Oocytes were injected with fluorescently labeled VLE RNA and cultured for 4–24 h before fixation and imaging. (A) At 4 h, the injected RNA is found predominantly in the perinuclear cup region, with little accumulation at the oocyte cortex. (B) By 8–10 h, the injected RNA is evident throughout the vegetal cytoplasm, with significant accumulation at the oocyte cortex. (C) Twenty-four hours after injection, the RNA is predominantly cortical, with little RNA in the vegetal cytoplasm and none detected in the cup region. Representative confocal images are shown, with the vegetal pole towards the bottom. Scale bars, 50 µm.
(TIF)

Figure S3 Inhibition of dynein by dynactin disruption does not alter microtubule organization in the vegetal cytoplasm. Oocytes were injected with (A) the CC1 domain of p150^glued^ of dynactin or (B) no exogenous protein (Control), and incubated overnight to allow exogenous protein expression. Microtubules were stained with anti-α-tubulin (Sigma) and fluorescent secondary antibodies before imaging by confocal fluorescence microscopy. Shown are representative confocal sections cropped to show the vegetal cytoplasm. Scale bars, 20 µm.
(TIF)

Figure S4 Inhibition of dynein by dynactin disruption does not alter dynein distribution in the vegetal cytoplasm. Oocytes were injected with (A) the CC1 domain of p150^glued^ of dynactin (CC1) or (B) no exogenous protein (Control), and incubated overnight to allow expression of exogenous protein. Dynein was detected with anti-dynein (DIC 74.1, Abcam) antibodies and fluorescent secondary antibodies before confocal microscopy. Shown are representative confocal sections cropped to show the vegetal cytoplasm. Scale bars, 50 µm.
(TIF)

Figure S5 Kinesin-1 Δmotor mutant disrupts vegetal RNA localization. (A) Uninjected oocytes or (B) oocytes injected with 250 nM kinesin-1 heavy chain lacking the motor domain (XKHCΔm [12]) were subsequently injected with fluorescently labeled VLE RNA and cultured for 8 h. Representative confocal images are shown, with the vegetal pole toward the bottom. Scale bars, 50 µm.
(TIF)

Figure S6 Transport directionality is not evident for RNAs that are not undergoing localization. Oocytes expressing PA-mCh-MCP were microinjected with (A) βG-MS2 RNA or (B–C) VLE-MS2 RNA. Time courses after activation in (A–B) the lower vegetal cytoplasm (Region 2) in untreated (A) and nocodazole-treated (B) oocytes or (C) the animal hemisphere cytoplasm (Region 4) are shown. Fluorescence intensities in the V (black) and A (grey) quadrants are shown. After activation (A–C), fluorescence returned to background levels within 20–30 s.
(TIF)

Figure S7 Averaged RNA transport directionality in specific regions. After activation of PA-mCh-MCP, as in Figure 5, the endpoint intensities were determined in the four collection quadrants (A, V, L, R) by taking an average of the quadrant intensity values over the last 20 time points (440–480 s of a 480 s time course) after activation in (A) the upper vegetal cytoplasm (*n* = 10 oocytes), (B) the lower vegetal cytoplasm (*n* = 10 oocytes), (C) the animal hemisphere (*n* = 19 oocytes), and (D) in nocodazole-treated oocytes activated in the vegetal cytoplasm (*n* = 13 oocytes). The value for a given quadrant was calculated as the percentage of total intensity (V+A or L+R), and all error bars indicate standard deviation. *p* values were generated using an unpaired Student's *t* test; *p = 0.00001, **p = 0.16.
(TIF)

Figure S8 Dynein remains colocalized with VLE RNA after localization. Oocytes injected with VLE RNA were incubated for 24 h to allow the majority of the injected RNA to complete localization, then probed with anti-dynein (DIC 74.1, Abcam) and fluorescent secondary antibodies before confocal microscopy. Shown is a confocal section: (A) VLE RNA and (B) dynein. A cropped and zoomed view (C) shows co-localization in the vegetal cytoplasm, with VLE RNA in red, dynein in green, and co-localization in yellow. Scale bars, 50 µm.
(TIF)

## Abbreviations

FRAP: fluorescence after photobleaching
RNP: ribonucleoprotein particle
UTR: untranslated region
VLE: vegetal localization element
