# Supplementary figures and images for: Directional Transport Is Mediated by a Dynein-Dependent Step in an RNA Localization Pathway
Source: PLoS Biol. 2013 Apr 30;11(4):e1001551. doi: 10.1371/journal.pbio.1001551 (PMC3640089; doi:10.1371/journal.pbio.1001551)

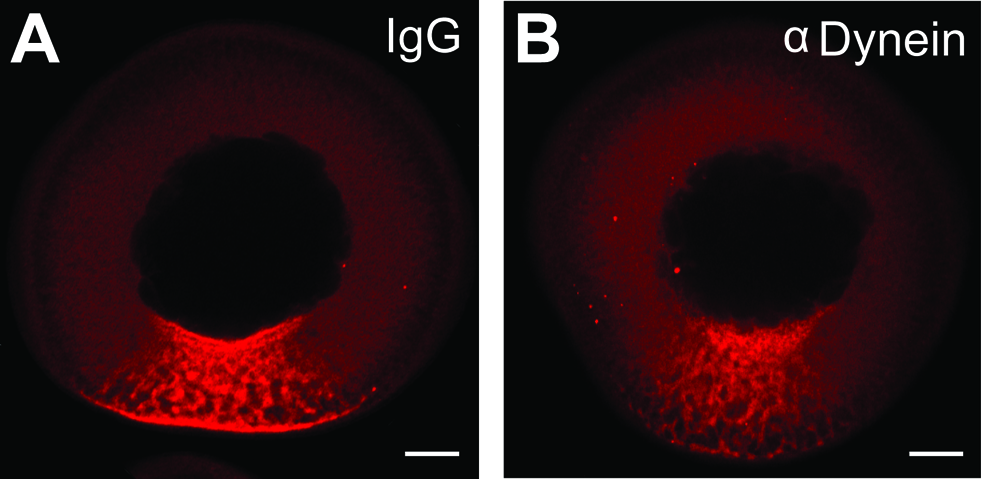

Supplement: Figure S1 — Dynein antibody injection blocks vegetal RNA localization. Oocytes were injected with either (A) control IgG (Sigma) or (B) function-blocking [53] dynein-specific antibodies (DIC 70.1, Abcam). After culture for 2 h, oocytes were injected with fluorescently labeled VLE RNA and cultured for 8 h. Oocytes were fixed and imaged by confocal microscopy. Representative confocal images are shown, with the vegetal pole toward the bottom. Scale bars, 50 µm. (TIF) [file pbio.1001551.s001.tif]

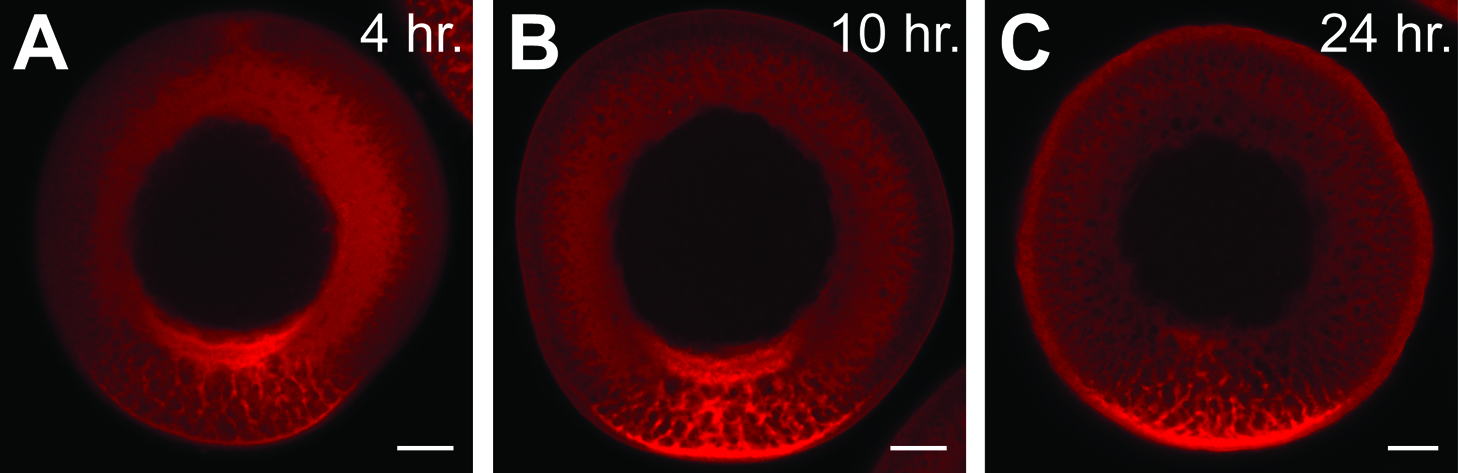

Supplement: Figure S2 — Time course of vegetal RNA localization. Oocytes were injected with fluorescently labeled VLE RNA and cultured for 4–24 h before fixation and imaging. (A) At 4 h, the injected RNA is found predominantly in the perinuclear cup region, with little accumulation at the oocyte cortex. (B) By 8–10 h, the injected RNA is evident throughout the vegetal cytoplasm, with significant accumulation at the oocyte cortex. (C) Twenty-four hours after injection, the RNA is predominantly cortical, with little RNA in the vegetal cytoplasm and none detected in the cup region. Representative confocal images are shown, with the vegetal pole towards the bottom. Scale bars, 50 µm. (TIF) [file pbio.1001551.s002.tif]

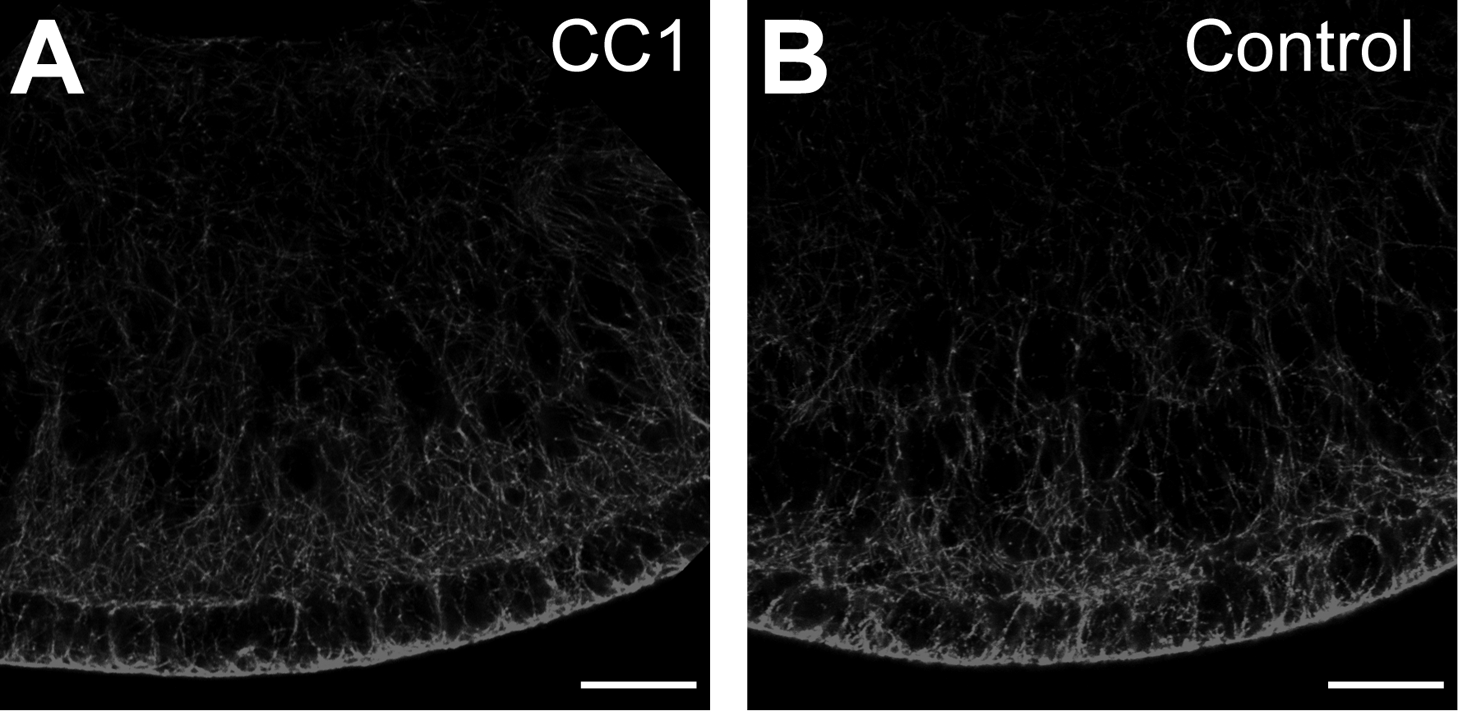

Supplement: Figure S3 — Inhibition of dynein by dynactin disruption does not alter microtubule organization in the vegetal cytoplasm. Oocytes were injected with (A) the CC1 domain of p150glued of dynactin or (B) no exogenous protein (Control), and incubated overnight to allow exogenous protein expression. Microtubules were stained with anti-α-tubulin (Sigma) and fluorescent secondary antibodies before imaging by confocal fluorescence microscopy. Shown are representative confocal sections cropped to show the vegetal cytoplasm. Scale bars, 20 µm. (TIF) [file pbio.1001551.s003.tif]

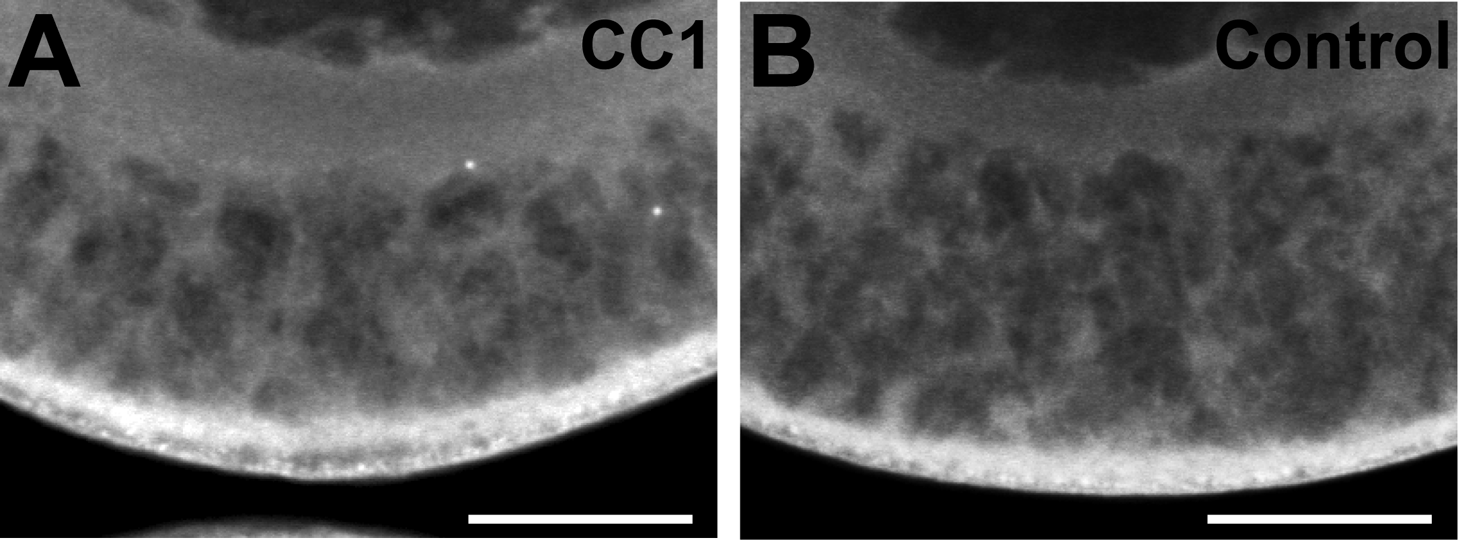

Supplement: Figure S4 — Inhibition of dynein by dynactin disruption does not alter dynein distribution in the vegetal cytoplasm. Oocytes were injected with (A) the CC1 domain of p150glued of dynactin (CC1) or (B) no exogenous protein (Control), and incubated overnight to allow expression of exogenous protein. Dynein was detected with anti-dynein (DIC 74.1, Abcam) antibodies and fluorescent secondary antibodies before confocal microscopy. Shown are representative confocal sections cropped to show the vegetal cytoplasm. Scale bars, 50 µm. (TIF) [file pbio.1001551.s004.tif]

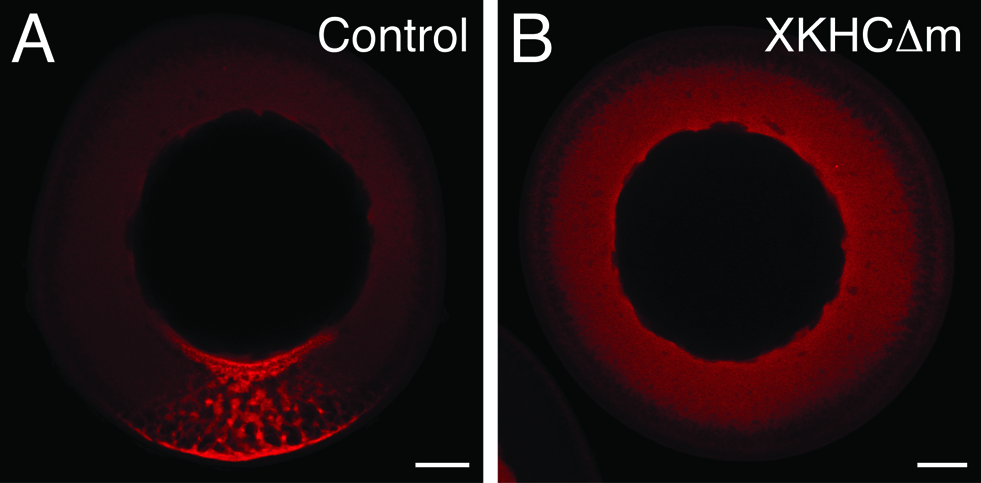

Supplement: Figure S5 — Kinesin-1 Δmotor mutant disrupts vegetal RNA localization. (A) Uninjected oocytes or (B) oocytes injected with 250 nM kinesin-1 heavy chain lacking the motor domain (XKHCΔm [12]) were subsequently injected with fluorescently labeled VLE RNA and cultured for 8 h. Representative confocal images are shown, with the vegetal pole toward the bottom. Scale bars, 50 µm. (TIF) [file pbio.1001551.s005.tif]

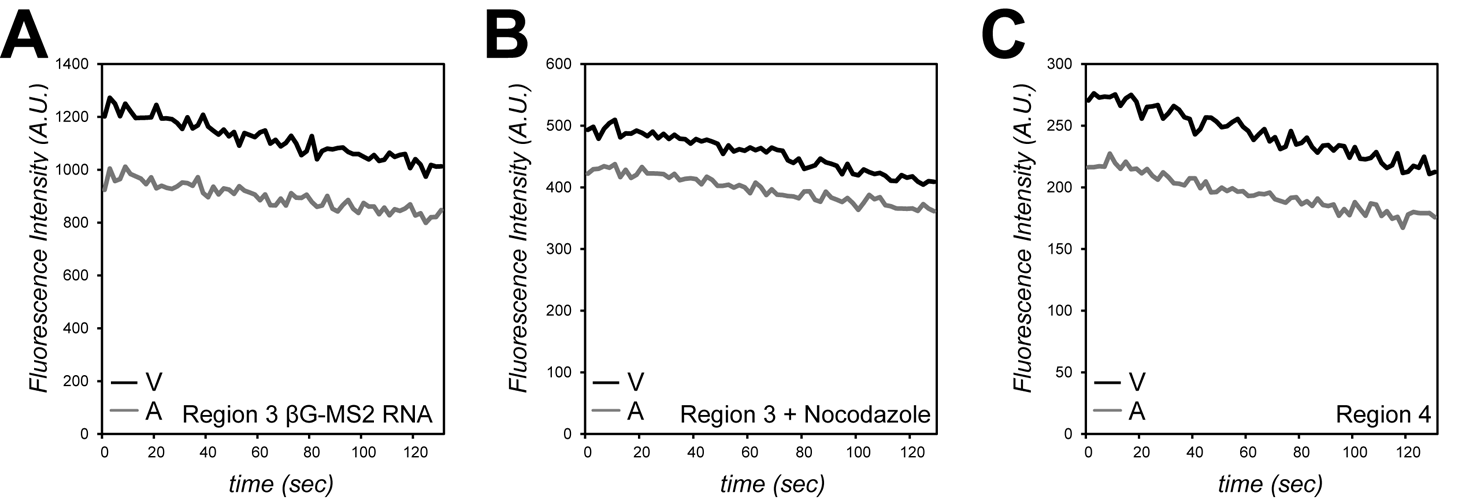

Supplement: Figure S6 — Transport directionality is not evident for RNAs that are not undergoing localization. Oocytes expressing PA-mCh-MCP were microinjected with (A) βG-MS2 RNA or (B–C) VLE-MS2 RNA. Time courses after activation in (A–B) the lower vegetal cytoplasm (Region 2) in untreated (A) and nocodazole-treated (B) oocytes or (C) the animal hemisphere cytoplasm (Region 4) are shown. Fluorescence intensities in the V (black) and A (grey) quadrants are shown. After activation (A–C), fluorescence returned to background levels within 20–30 s. (TIF) [file pbio.1001551.s006.tif]

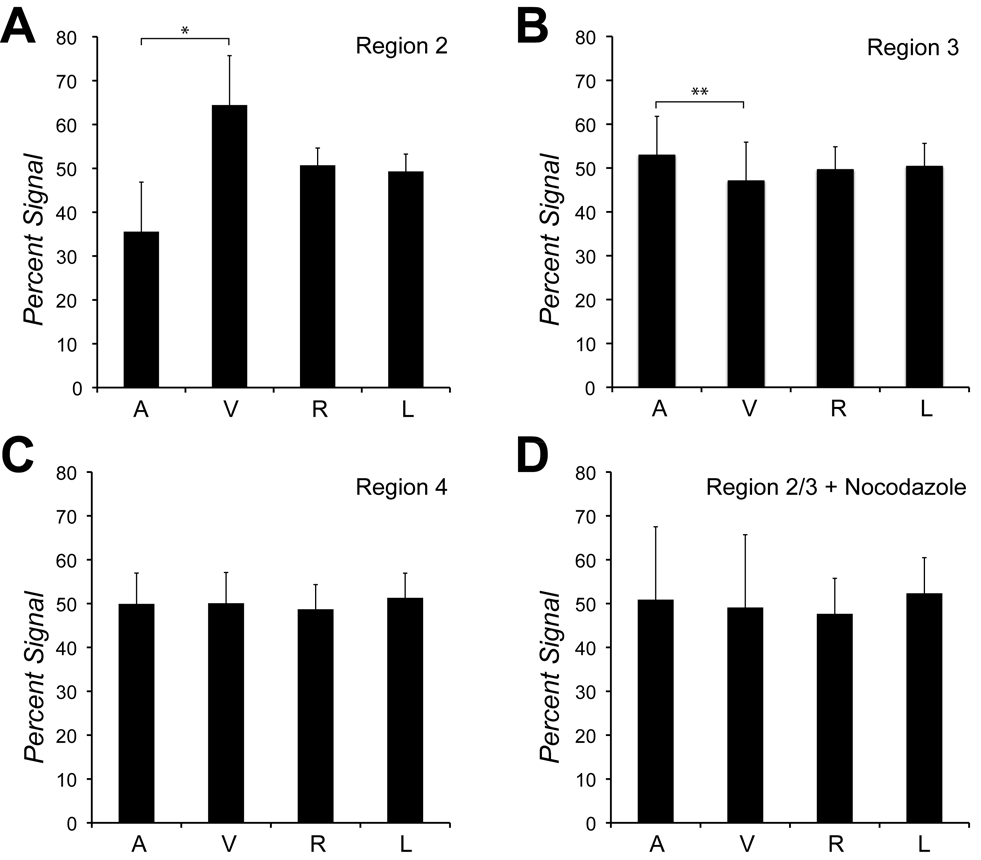

Supplement: Figure S7 — Averaged RNA transport directionality in specific regions. After activation of PA-mCh-MCP, as in Figure 5, the endpoint intensities were determined in the four collection quadrants (A, V, L, R) by taking an average of the quadrant intensity values over the last 20 time points (440–480 s of a 480 s time course) after activation in (A) the upper vegetal cytoplasm (n = 10 oocytes), (B) the lower vegetal cytoplasm (n = 10 oocytes), (C) the animal hemisphere (n = 19 oocytes), and (D) in nocodazole-treated oocytes activated in the vegetal cytoplasm (n = 13 oocytes). The value for a given quadrant was calculated as the percentage of total intensity (V+A or L+R), and all error bars indicate standard deviation. p values were generated using an unpaired Student's t test; *p = 0.00001, **p = 0.16. (TIF) [file pbio.1001551.s007.tif]

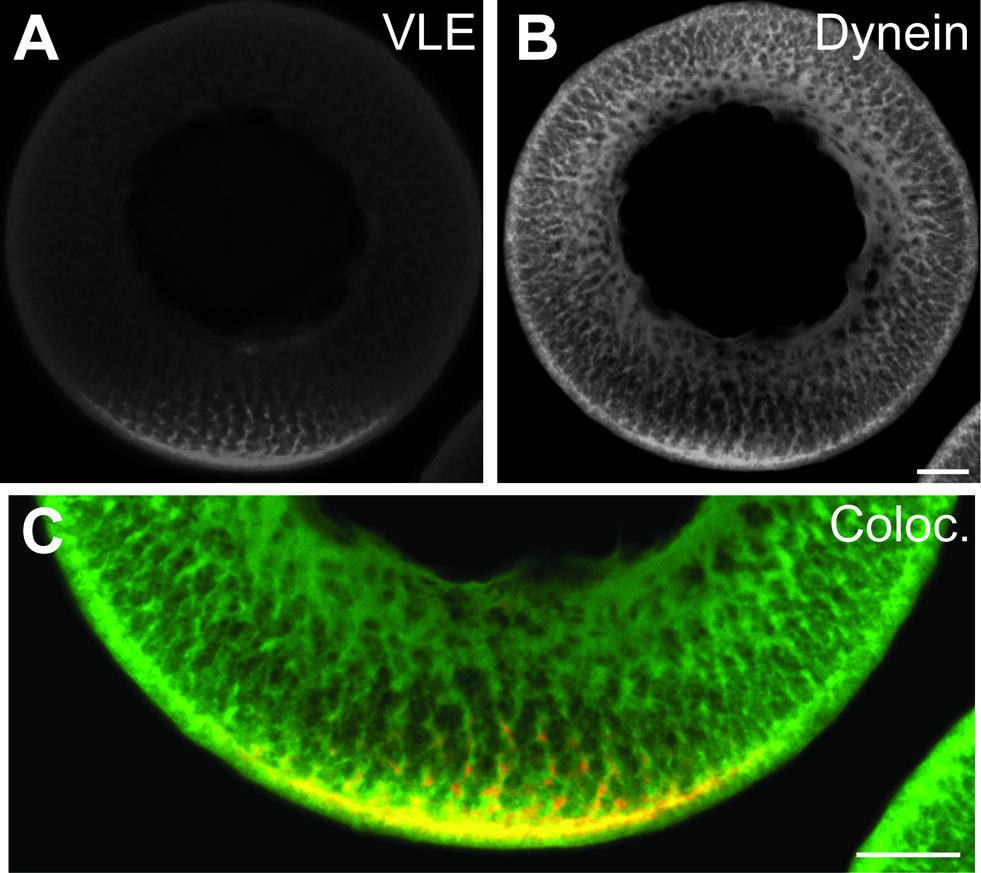

Supplement: Figure S8 — Dynein remains colocalized with VLE RNA after localization. Oocytes injected with VLE RNA were incubated for 24 h to allow the majority of the injected RNA to complete localization, then probed with anti-dynein (DIC 74.1, Abcam) and fluorescent secondary antibodies before confocal microscopy. Shown is a confocal section: (A) VLE RNA and (B) dynein. A cropped and zoomed view (C) shows co-localization in the vegetal cytoplasm, with VLE RNA in red, dynein in green, and co-localization in yellow. Scale bars, 50 µm. (TIF) [file pbio.1001551.s008.tif]
